# Microstructure and Mechanical Properties of High-Pressure Die-Casting Mg–Al–RE Alloys with Minor Ca Addition

**DOI:** 10.3390/ma18020231

**Published:** 2025-01-08

**Authors:** Sheng Guan, Pengyue Wang, Tianhua Wang, Chenggang Wang, Guojun Liu, Yongfu Zhu

**Affiliations:** 1 Key Laboratory of Automobile Materials, Ministry of Education, School of Materials Science and Engineering, Jilin University, 5988# Renmin Street, Changchun 130025, China; guansheng22@mails.jlu.edu.cn (S.G.); liuguojun@jlu.edu.cn (G.L.); 2 FAW Foundry Co., Ltd., Crossing of Hexie Street & Bingwu Road, Changchun 130013, China

**Keywords:** high-pressure die casting, Mg alloys, rare earth element, aging treatment, Mg-Al-RE

## Abstract

With the increasing demand for magnesium (Mg) alloys with high strength and good ductility, this study explores high-pressure die-cast (HPDC) Mg-6Al-2RE (AE62), Mg-8Al-2RE (AE82) and Mg-8Al-2RE-0.2Ca (AEX820) alloys (wt. %). Their microstructures and mechanical properties are investigated under both as-cast and T5-aged (direct artificial aging after casting) conditions. HPDC alloys consist of outer fine-grain regions and inner coarse α-Mg grains with abundant eutectic phases. The increasing addition of Al has an insignificant effect on the refinement of the grain size of α-Mg, but it significantly influences the morphology and area fraction of the second phases. The average grain sizes of α-Mg in HPDC AE62, AE82 and AEX820 alloys are approximately 4.0 μm, 3.9 μm and 3.7 μm in the edge regions and about 13.9 μm, 12.8 μm and 12.1 μm in the core regions, respectively. When aged at 200 °C, β-Mg_17_Al_12_ precipitates are predominantly formed in the studied alloys. Increasing the Al and Ca concentrations effectively refines the microstructures and enhances the aging hardening response and the strength, albeit at the expense of considerably reduced ductility. The peak-aged AE62 alloys demonstrate balanced tensile properties, with ultimate tensile strength (UTS), yield strength (YS), and elongation at fracture (E_f_) at room temperature of ~241 MPa, ~141 MPa and ~7.1%, respectively, and values of ~129 MPa, ~96 MPa and ~19.8%, respectively, at 175 °C. Compared to peak-aged AE62 alloy, the UTS and YS of peak-aged AEX820 alloys are improved by ~6.7% and ~14.2%, respectively, at RT and ~8.5% and ~12.5% at 175 °C, while E_f_ is decreased by 35.2% at RT and 33.3% at 175 °C, primarily due to the high area fraction of secondary phases.

## 1. Introduction

As energy shortages and environmental concerns become more pronounced, lightweight materials are becoming increasingly important in transportation [1,2,3]. Magnesium (Mg) alloys, known for their high specific strength, specific stiffness, excellent damping properties, degradability and recyclability, are widely used in the aerospace, transportation and medical industries [2]. Among different studies on Mg alloys, much attention has been paid to high-pressure die-cast (HPDC) Mg alloys, particularly those based on Mg-Al systems such as Mg-Al-Zn (AZ), Mg-Al-Mn (AM) and Mg-Al-RE (AE), which have been employed in consumer electronics (3C products) and automotive applications as non-structural components [4,5,6]. Nevertheless, current studies indicate that these HPDC Mg alloys still have some shortcomings, including low strength and high cost, limiting their wider application.

HPDC provides advantages such as rapid solidification rates, resulting in fine microstructures and enhanced strength compared to traditional casting methods [7], making it valuable for producing Al, Mg and Zn alloys [8,9,10]. Since the 1970s, significant efforts have been devoted to improving HPDC Mg-Al-RE alloys, which are seen as having significant potential [9,11,12,13]. Among these, Mg-4Al-4RE (wt.%; the same unit applies hereafter) alloy has received considerable attention [12]. For instance, tensile tests conducted by Lee et al. [14] on AE44 Mg alloy specimens revealed notable variations in ductility ranging from 7.1% to 13.1%, which were attributed to defects. Zhang et al. [15] further explored HPDC Mg-4Al-4RE-0.4Mn alloys, comparing the effects of mixed rare earth element additions. Their findings indicated that the combination of Ce and La improves tensile properties due to a refined grain structure and second phases, offering a cost advantage over the use of single rare earth elements. Another study by Su et al. [16] demonstrated that varying Al concentrations affect the properties of HPDC Mg-4Ce-0.5Mn alloys, concluding that 3% Al addition optimizes tensile properties due to fine second phases. Despite the promising tensile properties of HPDC AE44 alloys, their high cost, due to the 4% RE concentration, has shifted research focus towards developing HPDC AE42 series alloys with reduced rare earth concentrations and better mechanical properties [17,18]. However, these alloys face casting challenges due to lower alloying element concentrations, which easily cause hot tearing during HPDC processes. Therefore, further research into HPDC Mg-Al-RE alloys is still necessary.

A significant limitation of HPDC Mg alloys is their perceived unsuitability for heat treatment, primarily due to internal defects (e.g., gas pores and shrinkage) that may expand during heating, resulting in surface blistering or dimensional deformation [19]. To address this issue, two potential strategies could be considered. The first involves using high-vacuum conditions during the HPDC process to minimize porosity [20]. Although it is effective for Mg and Al alloys, this approach requires expensive and complex equipment. The alternative is a modified heat treatment involving lower temperatures and a shorter time (T5 aging treatment), avoiding high-temperature solution treatment. This direct T5 aging process has been successfully applied to HPDC Mg-2.70La-1.50Y alloys without inducing surface blistering or dimensional instability [21]. Zhu et al. [22] found that the tensile strength of HPDC Mg-4Al-3La-0.3Mn alloy is enhanced by direct aging treatment at 200 °C, attributed to nano-scale Al-Mn precipitates. A study by Pettersell et al. [23] also highlighted significant peak-aged micro-hardness in HPDC AE41 alloys subjected to T5 treatment at various temperatures. This modified method is effective for enhancing the strength of die-cast Al-Mg-Si alloys, indicating its potential as a viable approach for improving tensile properties of HPDC Mg alloys [3,24]. However, further research into HPDC Mg-Al-RE alloys remains scarce.

Alloying plays a crucial role in enhancing the properties of Mg alloys. Al, as a predominant alloying element, improves casting performance up to a certain level; however, when its concentration is below 6%, it can reduce fluidity and castability. Conversely, exceeding 9% Al makes Mg alloys brittle and decreases performance [25]. In contrast, studies of concentrations ranging from 7 to 9% have shown that 8% Al in Mg-1.5Ce-0.7Zn alloys yields optimal results [26]. Ca is also one alloying element that plays a role in enhancing cleanliness and creep performance in AZ91 alloy, although levels above 0.3% in AM50 alloys lead to the increased shrinkage and porosity [5,27]. Based on the above discussion, Mg-6Al-2RE, Mg-8Al-2RE and Mg-8Al-2RE-0.2Ca alloys were designed and studied by investigating their microstructures, phase compositions and mechanical properties under both die-cast and peak-aged conditions. This exploration aims to enhance the effectiveness and applicability of HPDC Mg alloys in addressing current challenges in transportation and beyond.

## 2. Materials and Methods

**Figure 1:**
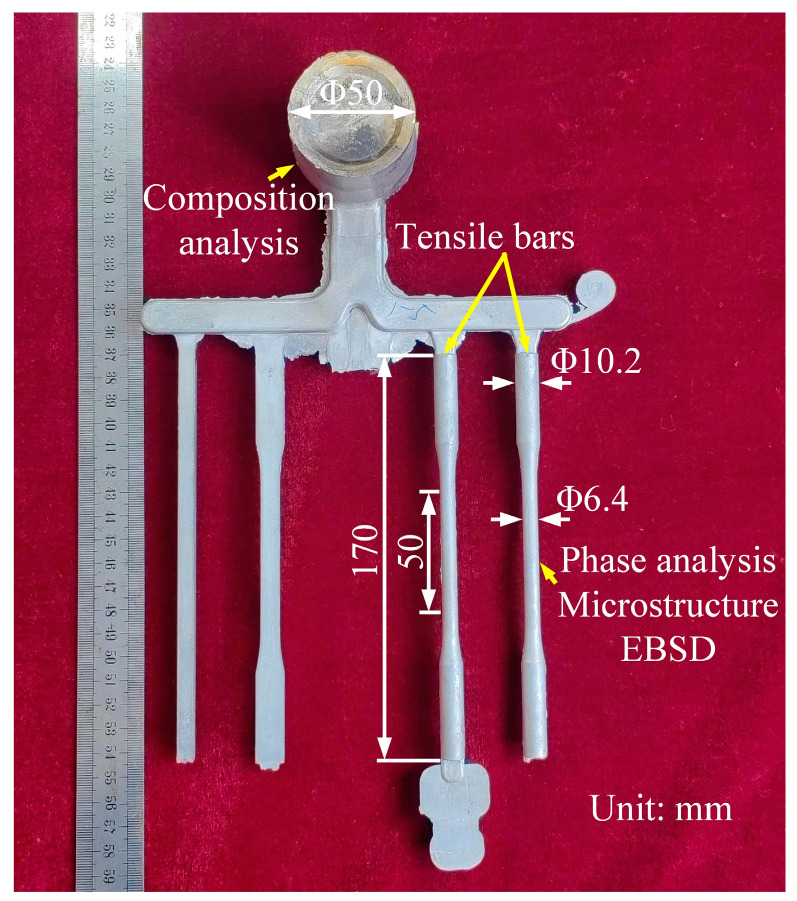
Image of the studied HPDC die-casting product with dimensions of tensile bars and sample regions.

The nominal compositions of Mg-6Al-2RE, Mg-8Al-2RE and Mg-8Al-2RE-0.2Ca (wt. %) alloys were prepared using pure Mg (99.9%), Al (99.9%), and master alloys of Mg-30%RE (Ce-rich Ce-La rare earth mixtures) and Mg-30%Ca. These alloys are hereafter referred to as AE62, AE82 and AEX820, respectively. A total of 40 kg of raw materials were fully melted at approximately 700 °C in a low steel crucible using a resistance furnace under a protective atmosphere of N_2_ and SF_6_ gases. The molten metal was maintained at around 690 °C for 30 min before proceeding with high-pressure die casting (HPDC). The HPDC process was conducted using an IMPRESS DCC280T cold-chamber machine (L.K. Group, Ningbo, China) with a steel mold equipped with an oil heating/cooling system to control the die temperature, which was set at 280~300 °C. The specific parameters used in this study were as follows: a pouring temperature of 670~690 °C, injection pressure of 30~40 MPa and die holding time of 3~4 s. The appearance and dimensions of the HPDC samples are shown in Figure 1, where the regions of samples and the tensile bars can be observed. The aging treatment (T5) of the tensile bars was performed at 200 °C for 24 h in a silicone oil bath followed by air cooling.

All polished metallographic specimens were etched with Nital (a solution of 4% nitric acid) for 5~10 s. Samples used for electron backscatter diffraction (EBSD) investigation were electro-polished in commercial AC2 solution (20 V, ~90 s) after grounding with 1000, 2000 and 3000 mesh SiC sand papers in turn and polishing with 0.5 μm diamond abrasion paste, followed by cleaning with absolute ethanol. Thin foils used for transmission electron microscopy (TEM) examinations were prepared by mechanical polishing to a thickness of ~80 μm, followed by the punching of discs of 3 mm in diameter and ion-beam milling using a Gatan PIPS 691 at 5 keV and ~−100 °C.

The actual chemical compositions of HPDC samples were verified using inductively coupled plasma–atomic emission spectrometry (ICP-AES, Plasma 3000 ICP, The NCS Testing Technology Co., Ltd., Kunshan, China), with results listed in Table 1. The phase constituents of the samples were examined using X-ray diffraction (XRD, DX-2700B, Dandong Haoyuan Instrument Co., Ltd., Dandong, China) with Cu Kα radiation, conducting 2θ scans from 20° to 80° at a speed of 2°/min. The microstructures were investigated using a field emission scanning electron microscope (FESEM, JSM-7900, JEOL, Tokyo, Japan) equipped with an energy-dispersive spectrometer (EDS, INCA-X-Max, Oxford Instruments, High Wycombe, UK) and an electron backscatter diffraction (EBSD, Oxford Instrument, High Wycombe, UK) detector with Aztec 3.0 software. EBSD was conducted at 15 kV with a tilt angle of 70° and a scan step of 0.6 μm. The EBSD data were analyzed using HKL Channel5 software (version 5.12.73.0, Oxford Instruments, High Wycombe, UK), with results reported as the mean grain size distribution of α-Mg [28,29]. The peak-aged samples were characterized using transmission electron microscopy (TEM, TalosF200X, FEI, Hillsborough, OR, USA). The area fraction of the secondary phases of HPDC samples was quantified from the backscattered electron (BSE) images on a FESEM using Image Pro Plus 6.0 software.

Tensile tests were conducted with a strain rate of 1.0 × 10^−3^ s^−1^ at room temperature (RT) and 175 °C using a materials testing system (MTS, MTS-810, Minneapolis, MN, USA). The gauge length of tensile bars was 50 mm, with a diameter of 6.4 mm (Figure 1). The holding time of specimens was 10 min before tensile testing at 175 °C. At least three specimens of each alloy were subjected to testing under specific conditions. The micro-hardness of samples was investigated using a Vickers hardness tester with a load of 0.49 N for 15 s. The average micro-hardness values were calculated from 10 individual indentations, excluding the maximum and minimum values.

## 3. Results and Discussion

### 3.1. The Phase Compositions and Microstructures of as-Cast Alloys

**Figure 2:**
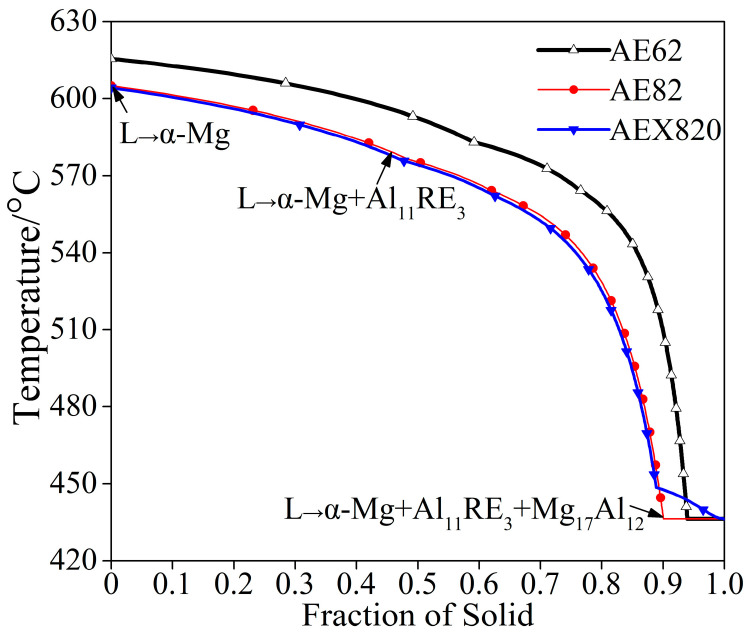
Scheil simulation results of solid fraction vs. temperature for studied alloys.

It is widely recognized that not all Mg alloys are suitable for HPDC [2]. In the design of HPDC Mg alloys, one of the primary considerations is the solidification range [7]. Both excessively narrow and excessively wide solidification intervals are detrimental to successful HPDC processing [30]. To investigate the solidification characteristics of the studied Mg alloys, phase diagrams were calculated utilizing the latest Pandat Mg database and non-equilibrium (Scheil’s) modeling based on computational thermodynamics [31]. As illustrated in Figure 2, the solidification ranges for AE62, AE82 and AEX820 alloys are 179.32 °C, 168.84 °C and 168.10 °C, respectively, from the onset to the completion of solidification. These solidification ranges reflect a slight decrease compared to the AE44 alloy’s range of 185.93 °C, indicating that the designed alloys are suitable for HPDC, given the rapid cooling rates characteristic of the die-casting process for Mg alloys.

The solidification sequences of the studied alloys, which reveal complete mixing in the liquid phase without diffusion in the solid phase [32], are similar for these alloys despite variations in alloying element concentration. The calculated results (Figure 2) show that the phases are composed of α-Mg (hexagonal, P63/mmc space group), Al_11_RE_3_ (orthorhombic, Immm space group) and Mg_17_Al_12_ (cubic, I-43m space group) phases [33]. Taking AE82 as an example, the solidification path unfolds as follows: Initially, at the inception of solidification, solid phases begin to precipitate, specifically through the nucleation and growth of primary α-Mg (L→α-Mg, at 605 °C). Subsequently, a binary eutectic reaction occurs (L→α-Mg + Al_11_RE_3_, at 576 °C). Finally, as the solidification temperature continuous to decline, a ternary eutectic reaction takes place (L→α-Mg + Al_11_RE_3_ + Mg_17_Al_12_, at 436 °C), triggering an augmentation in the solid-phase fraction. Notably, Ca-containing phases were not observed in the simulated results for the AEX820 alloy, despite the introduction of 0.2% Ca. This absence may be attributed to its low concentration and the limitations of the Pandat software database.

**Figure 3:**
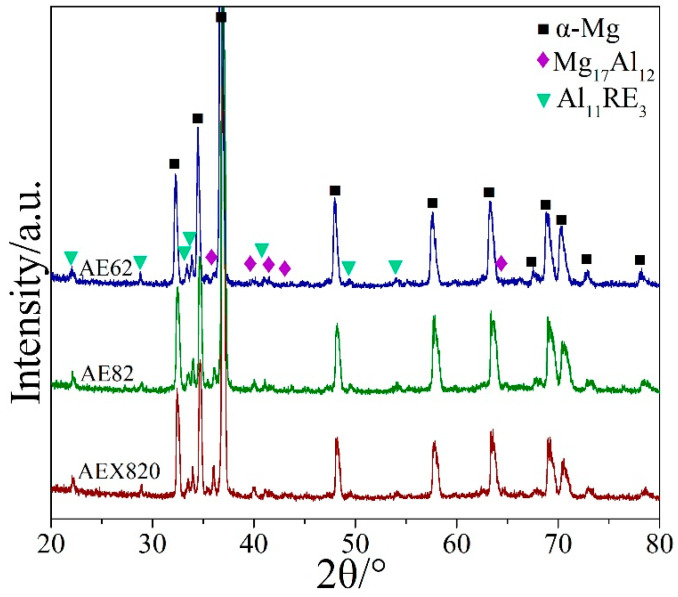
XRD patterns of HPDC samples.

Figure 3 presents the XRD patterns of the studied HPDC alloys. All samples comprise α-Mg, Mg_17_Al_12_ (β) and Al_11_RE_3_ (γ) phases, affirming that increasing Al does not alter the phase composition. The formation of a γ phase instead of a Mg-RE phase is attributed to the stronger electronegativity between Al and RE elements, which is greater than that between Mg and RE in Mg-Al-RE alloys [17,18]. These phases align with the predicted results deduced from phase-diagram calculation (Figure 2). The absence of Ca-containing phases is possibly attributable to the limited Ca addition relative to the Al_2_Ca phase in Mg-Al-Ca alloys [34].

**Figure 4:**
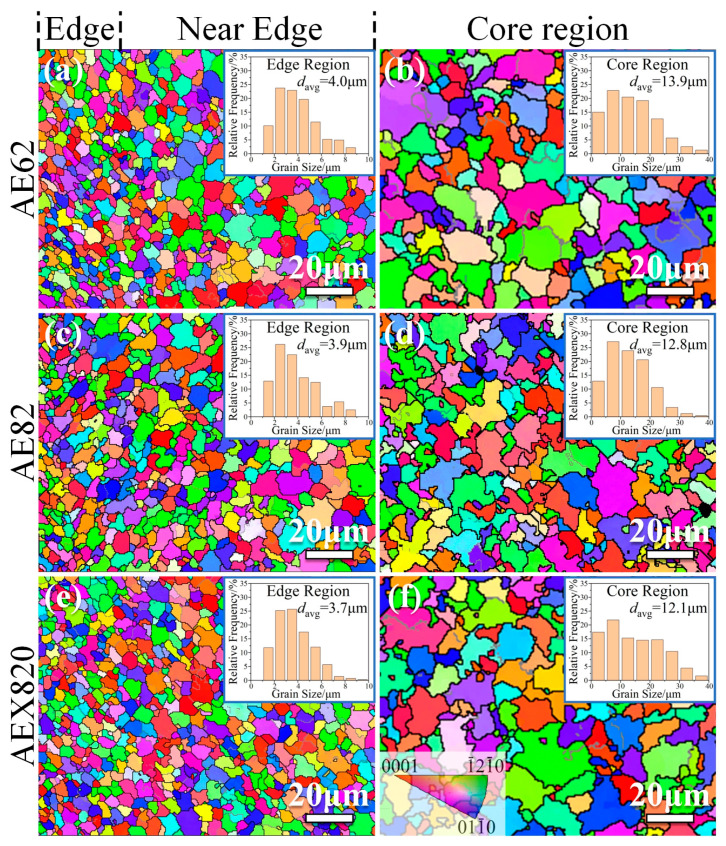
EBSD inverse pole diagram (IPF) of grain orientation and grain-size distributions of α-Mg in HPDC alloys. Note that the inserts in (**a**,**c**,**e**) are the grain-size distributions of α-Mg in the edge regions, whereas the inserts in (**b**,**d**,**f**) represent the grain-size distributions of α-Mg in the core regions. Additionally, the inset in the bottom left corner of (**f**) displays an inverse pole figure with codes representing the grain orientation.

Figure 4 illustrates the inverse pole figure (IPF) and the corresponding grain-size distribution of α-Mg in the HPDC alloys covering the edge region and core regions. The grain orientations in the samples manifest randomness, devoid of the preferred orientation typically observed in deformed Mg alloys [9]. It is evident that the average grain size in the edge region is small compared to the core region, with a progressive increase in grain size from the edge region to the core region. Statistical results reveal that the average grain sizes of α-Mg in the edge regions of the HPDC AE62, AE82 and AEX820 alloys are approximately 4.0 μm, 3.9 μm and 3.7 μm, respectively. Correspondingly, the average grain sizes in the core regions are about 13.9 μm, 12.8 μm and 12.1 μm, respectively, where the AEX820 alloy exhibits the smallest average grain size of α-Mg (Figure 4). This suggests that, as the alloying element concentration increases, the average grain size of the alloys tends to decrease. However, the trend of grain-size reduction becomes less pronounced from AE82 to AEX820 alloys.

The solidification range of AE62 is wider than that of the others, indicating that a slightly slower solidification process is still a little slow in the core regions, leading to relatively coarse α-Mg grains. Comparing AEX820 to the AE82 alloy, the enrichment of Ca at the solidification front caused by solute redistribution leads to stronger constitutional super-cooling during the solidification process of α-Mg dendrites [17,18]. This condition promotes the development of more branched α-Mg structures, which appear somewhat finer in AEX820 alloy compared to AE82 alloy, consistent with results reported for die-cast AZ91D Mg alloys [5].

**Figure 5:**
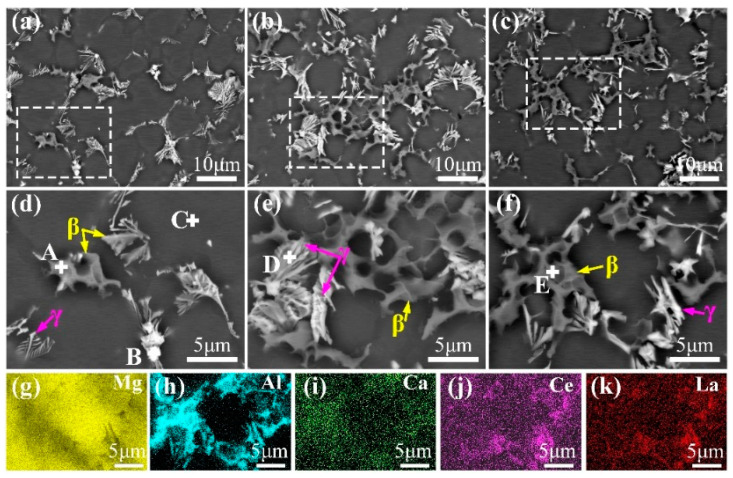
BSE images of studied HPDC alloys (**a**–**f**) and EDS mapping results of AEX820 alloy (**g**–**k**). (**a**,**d**) AE62; (**b**,**e**) AE82; (**c**,**f**) AEX820; (**d**–**f**) high-magnification photos of local regions marked by the white dotted lines in (**a**–**c**), respectively.

Figure 5 shows BSE images and EDS results for the core regions of HPDC samples. The core region’s secondary phases predominantly comprise gray net-broken-like phases; feather-like phases; and a few white, blocky phases (Figure 5a–f). Furthermore, EDS analyses conducted on points A to E, as detailed in Table 2, reveal that the gray discontinuous phase at point “A” is identified the β phase (Figure 5d); the white, blocky phase at point “B” is the Al_2_RE phase (Figure 5d) [18]; the phase at point “C” is the α-Mg phase (Figure 5d); the white, lamellar phase at point “D” is the γ phase (Figure 5e); and point “E” corresponds to a mixture of β and Al_2_Ca phases (Figure 5f).

Combining the XRD patterns (Figure 3) with the EDS results (Figure 5), it is deduced that the secondary phases in the core regions are primarily composed of the β phase (indicated by yellow arrows), γ phase (shown by pink arrows) and the Al_2_RE phase near point “B”. Unlike AE62, the β phase is coarser in the AE82 and AEX820 alloys due to their higher Al concentrations. Furthermore, the β phase in the AEX820 alloy is slightly smaller in size compared to the AE82 alloy, which is potentially attributable to the addition of Ca. This narrows the solidification range and accelerates the cooling rate. Note that the addition of Ca was found to be distributed mainly along the β phase. Statistical analysis indicates that the area fractions of secondary phases in AE62, AE82 and AEX820 alloys are approximately 14.1%, 18.7% and 19.8%, respectively.

**Figure 6:**
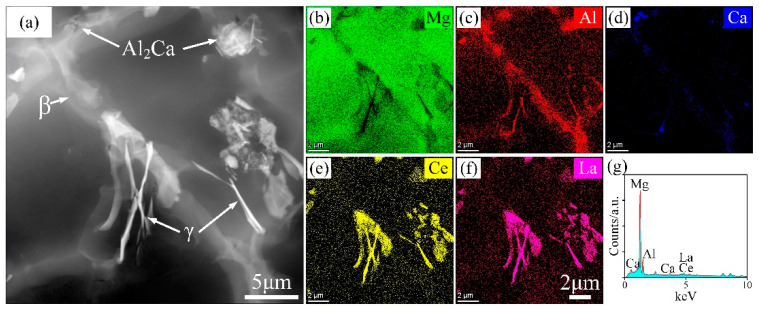
HAADF image of HPDC AEX820 alloy (**a**) and the EDS mapping results (**b**–**g**) on (**a**).

As to whether an Al_2_Ca phase was formed, the high-angle annular dark-field (HAADF) image of the AEX820 alloy is provided in Figure 6. It shows strong Ca signal intensities in small, localized regions, indicating the formation of a fine Al_2_Ca phase during the solidification process, primarily due to the high cooling rates caused by the HPDC processes [4]. Additionally, fine β and γ phases were also identified. Thus, from Figure 4, Figure 5 and Figure 6, it can be concluded that the microstructures of HPDC Mg alloys primarily exhibit two typical regions: an edge region composed of fine α-Mg grains and a core region consisting of relatively coarse α-Mg grains and abundant secondary phases, similar to the previous reports on HPDC alloys [17,18]. The addition of minor amounts of Ca induces few fine Al_2_Ca phases formed along the grain boundaries.

### 3.2. Age-Hardening Response of HPDC Alloys During T5 Aging Treatment

**Figure 7:**
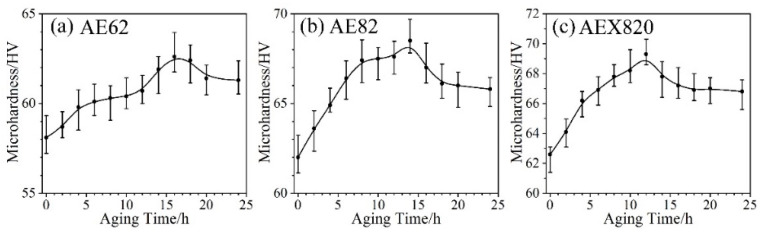
Micro-hardness vs. aging time curves of HPDC alloys during T5 aging treatment. (**a**) AE 62; (**b**) AE 82; (**c**) AEX820.

Figure 7 illustrates the relationship between micro-hardness and aging time for AE62, AE82 and AEX820 alloys during T5 aging treatment at 200 °C for 24 h. The micro-hardness initially increases, followed by a gradual decrease as the aging time increases, consistent with the typical micro-hardness evolution trend observed in aged Mg alloys [17]. Generally, in the early stages of aging treatment, the precipitates are relatively small and uniformly distributed, which contributes to the increase in micro-hardness. However, as aging time extends, the precipitates coarsen, resulting in a subsequent decrease in micro-hardness. Moreover, the peak micro-hardness and the time required to reach it differ significantly among the T5-treated alloys: 62.6 HV at 16 h, 68.5 HV at 14 h and 69.3 HV at 12 h for the AE62, AE82 and AEX820 alloys, respectively. This indicates that increasing the Al and Ca concentrations enhances the aging–hardening response. The variation in peak-aged micro-hardness is primarily related to the quantity and distribution of the fine precipitates formed during the T5 treatment process [35].

### 3.3. Microstructures of Peak-Aged Samples

**Figure 8:**
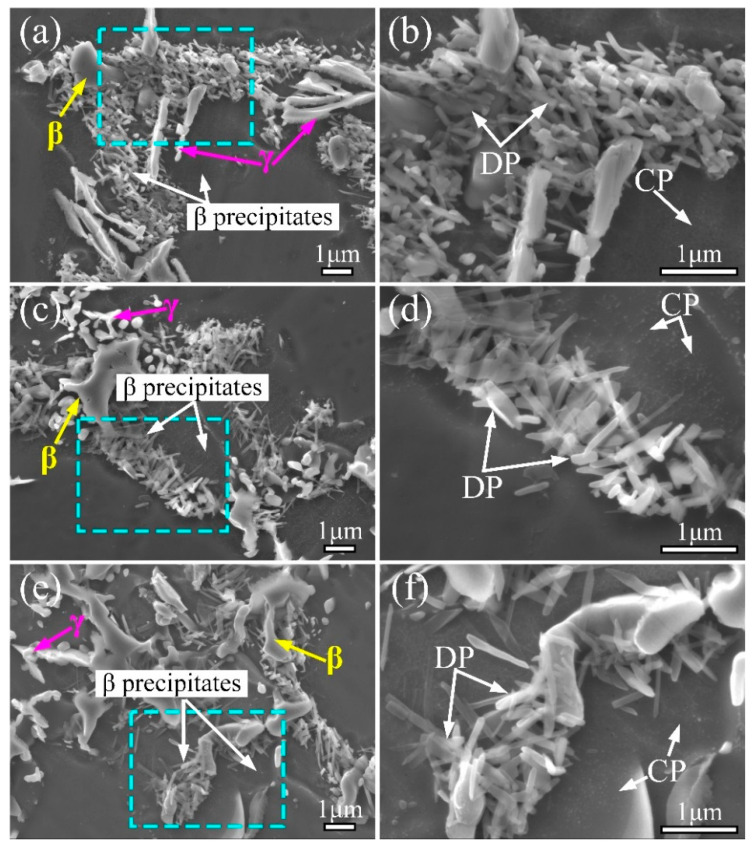
FESEM microstructures of the peak-aged alloys. (**a**,**b**) AE62; (**c**,**d**) AE82; (**e**,**f**) AEX820. Note that (**b**,**d**,**f**) are the high-magnification photos of the regions surrounded by the cyan dotted lines on (**a**,**c**,**e**), respectively. CP indicates continuous precipitate, while DP represents discontinuous precipitate.

Figure 8 presents the FESEM images of the microstructures of the peak-aged alloys. For the peak-aged AE62 alloy, it is evident that numerous precipitates are located at grain boundaries and within the grain interiors, along with the remaining large β and γ eutectic phases (Figure 8a). Further careful observation reveals that relatively coarse lamellar-like β precipitates are distributed along the grain boundaries, while “particle-like” β precipitates are sparse in the grain interiors (Figure 8a,b). This suggests that the precipitation of β precipitates is primarily controlled by discontinuous precipitates (DPs) rather than continuous precipitates (CPs) during the T5 aging treatment [36]. With increasing Al and Ca contents, the fraction of lamellar-like β precipitates along the grain boundaries in the AE82 and AEX820 alloys significantly decreases (Figure 8c,e), while the number of particle-like β precipitates within the grain interiors increases (Figure 8d,f) compared to those in AE62 (Figure 8a,b). This increase in CPs is beneficial for enhancing the aging response and the strength of the peak-aged alloys.

**Figure 9:**
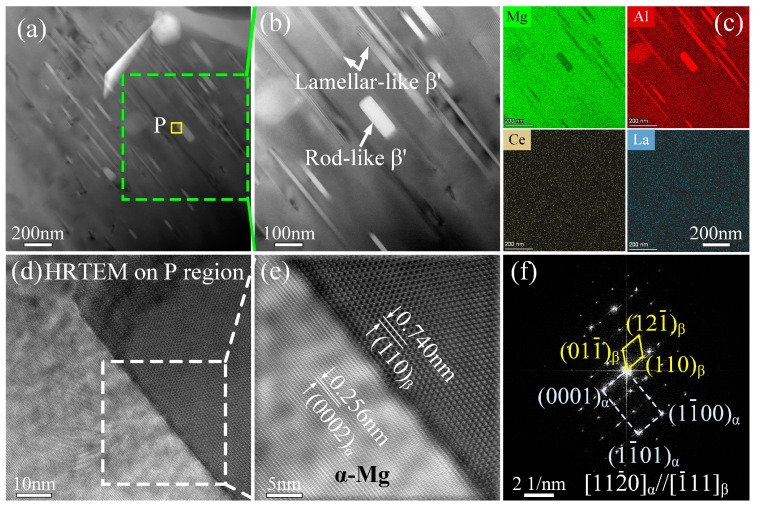
TEM investigation of peak-aged AE62 alloy. (**a**) HAADF image of peak-aged AE62 alloy; (**b**) the local high-magnification HAADF image at the regions surrounded by the green dotted lines; (**a**,**c**) STEM-EDS results; (**b**,**d**) HRTEM in P the region; (**a**,**e**) IFFT image of the local region in (**d**) outlined by the white dotted lines; (**f**) the FFT patterns in (**e**). β′ represents β precipitates. The electron beam is parallel to [11–20]_α_.

To further characterize these precipitates, TEM observation was performed on these specimens, with the typical images of AE62 presented in Figure 9. The HAADF image reveals a substantial number of precipitates (Figure 9a). The high-magnification HAADF image of the local region outlined by the green dotted lines in Figure 9a clearly shows a predominance of gray, lamellar-like and a few bright, rod-like precipitates (Figure 9b). Moreover, EDS analysis results (Figure 9c) confirm that these phases are β-Mg_17_Al_12_ precipitates (β′) [37]. Nie [35] reported that most continuous precipitates and discontinuous β-phase precipitates in aged Mg-Al serial alloys typically exhibit a lamellar structure. Consistent with the microstructures of the peak-aged AE62 alloy shown in Figure 8a,b, these fine β precipitates belong to continuous precipitates, which are more effective in enhancing mechanical properties compared to coarser, discontinuous precipitates typically found along grain boundaries [38]. Previous studies have indicated that rod-like precipitates are more effective than lamellar-like precipitates in impeding dislocation glide on the basal plane, although only a small fraction of rod-like precipitates is observed in Figure 9b [39]. To further investigate the rod-like precipitates, high-resolution transmission electron microscopy (HRTEM), inverse fast Fourier transform (IFFT) and fast Fourier transform (FFT) were employed to analyze the orientation relationship between α and β precipitates in the P regions marked by yellow lines in Figure 9a (Figure 9d–f). The interplanar spacing of β precipitates measured in the IFFT image (Figure 9e) is approximately 0.740 nm, closely matching the (110)_β_ spacing of 0.745 nm according to the Mg_17_Al_12_ PDF card (PDF#97-015-8247), indicating that the interplanar spacing corresponds to (110)_β_. Similarly, the interplanar spacing of 0.256 nm corresponds to (0002)_Mg_ based on the Mg PDF card (PDF#97-007-6748). The orientation relationship between α and β precipitates, determined by indexing the diffraction spots in the FFT image (Figure 9f), is (0001)_α_//(110)_β_ and [11–20]_α_//[-111]_β_ according to the indexing of diffraction spots in the FFT image (Figure 9f). This orientation relationship is consistent with the typical Burgers relationship commonly observed in aged Mg-Al alloys [35,38]. These findings suggest that mechanical property enhancement is limited because β precipitates aligned along the basal plane are less effective in impeding dislocation glide compared to those along the prism or pyramidal planes of α-Mg.

### 3.4. Tensile Properties and Fracture Behaviors

**Figure 10:**
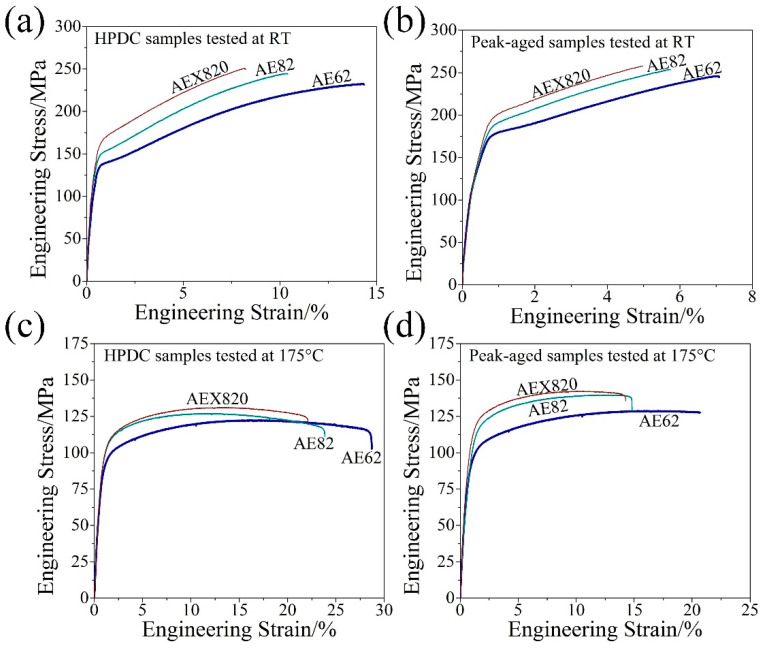
The engineering stress–stain curves of HPDC and peak-aged alloys tested at RT and 175 °C. HPDC specimens tested at RT (**a**) and 175 °C (**c**); peak-aged alloys tested at RT (**b**) and 175 °C (**d**).

Figure 10 illustrates the engineering stress–strain curves of both as-cast and peak-aged alloys at RT and 175 °C, respectively. The average values of the tensile properties are detailed in Table 3. At RT, the strength of both as-cast and peak-aged samples rises with increasing Al and Ca; however, the corresponding ductility exhibits a decreasing trend. As depicted in Figure 10a, the YS of the as-cast AEX820 alloys is notably higher than that of the AE82 and AE62 alloys at RT. Nonetheless, its ductility, measured at approximately 8.2%, which is significantly lower compared to the 9.8% for AE82 and 13.9% for AE62. Consequently, when considering the combined attributes of strength and ductility, the as-cast AE62 alloys exhibit superior tensile properties at RT, with ultimate tensile strength (UTS), yield strength (YS) and elongation at failure (E_f_) values of 232 MPa, 132 MPa and 13.9%, respectively. The room-temperature strength of AE62 was substantially enhanced following T5 aging treatment, with the elongation decreasing from 13.9% in the as-cast samples to 7.1% in the peak-aged samples (Figure 10b). This reduction in ductility is still notably higher than the 5.6% observed in AE82 and the 4.6% in AEX820 alloys (Figure 10b).

Upon increasing the tensile testing temperature to 175 °C, both the UTS and YS of the as-cast and peak-aged alloys decreased in comparison to their RT values (Figure 10). The aging treatment further augmented the strength of HPDC alloys at 175 °C at the expense of reduced ductility (Figure 10c,d). It is noteworthy that the peak-aged AE62 alloy still demonstrates considerable ductility at 175 °C, with an E_f_ of 19.8%, which is significantly higher than that of AE82 (14.3%) and AEX820 (13.2%) alloys. Although the high-temperature strength of AE82 and AEX820 alloys is slightly higher than that of AE62, the improvement is marginal, accompanied by a more substantial decrease in E_f_. Ductility is a crucial factor, particularly for die-cast Mg alloys, where connectivity performance is essential [3]. Therefore, considering the combined attributes of strength and ductility, the peak-aged AE62 alloy demonstrates superior tensile properties at 175 °C, with UTS, YS and E_f_ values of 129 MPa, 96 MPa and 19.8%, respectively.

It is widely recognized that the primary strengthening mechanisms of YS for as-cast Mg alloys include grain-refinement strengthening (*σ*_gr_), solid-solution strengthening (*σ*_ss_) and secondary-phase strengthening (*σ*_sp_) [36,40]. Consequently, the YS (*σ*_y_) of HPDC alloys at RT can be determined using Formula (1) [34]:

*σ*_y_ = *σ*_0_ + *σ*_gr_+ *σ*_ss_+ *σ*_sp,_ (1)

where *σ*_0_ represents the shear stress required for dislocation slip on the slip plane and is set at 12 MPa [41]. As previously mentioned, the HPDC tensile bars are composed of an outer layer of thin, fine-grained regions and an inner core of coarse-grained regions. It is also noteworthy that the grain size of α-Mg in the edge regions is very close. Therefore, the subsequent discussion primarily focuses on the microstructures of the core regions, which are the pivotal areas in HPDC alloys.

First, grain-refinement strengthening can be quantified using the Hall–Petch equation,

*σ*_gr_ = *k·d*^−1/2^, (2)

in which *k* is the Hall–Petch coefficient, approximated at 280 MPa·μm^1/2^, and *d* represents the average grain size of α-Mg [42]. After substituting the relevant parameters and grain sizes into Formula (2), the calculated *σ*_gr_ values are 75 MPa, 78 MPa and 81 MPa for AE62, AE82 and AEX820 alloys, respectively.

Secondly, as Al is the main solid-solution element in the studied HPDC alloys, the solid-solution strengthening can be estimated according to the following relationship [43]:

(3) $$\sigma_{\mathrm{ss}}={\left(\sum_{i}k_{i}^{1/n}C_{i}\right)}^{n},$$

where n is set to as 2/3 and the solid-solution strengthening coefficient for Al (*k*_Al_) is taken as 196 MPa (at.%) ^−2/3^, while the Al concentration in the matrix of AE62, AE82 and AEX820 alloys, as determined by EDS analysis, is 2.4, 3.2 and 3.0 at.%, respectively. The corresponding calculated values of *σ*_ss_ are 16 MPa, 20 MPa and 19 MPa, respectively.

Thirdly, the secondary phase significantly affects the mechanical properties of alloys, primarily through its distribution, size and fraction. The strengthening attributed to the secondary phase (σsp) can be assessed using the following equation [35]:

*σ*_sp_ = 4ϕγμε*f, (4)

where *ϕ* = μ*/(μ* *−* *γ*(μ* *−* *μ*)) and *μ* and μ* are the shear moduli of Mg and the second phase respectively; *γ* = 1/2(1 − *ν*) is an adjustment factor, with ν representing the Poisson ratio; ε* is the true strain plasticity, often considered as 0.2%; and *f* is the area fraction of the second phases.

**Figure 11:**
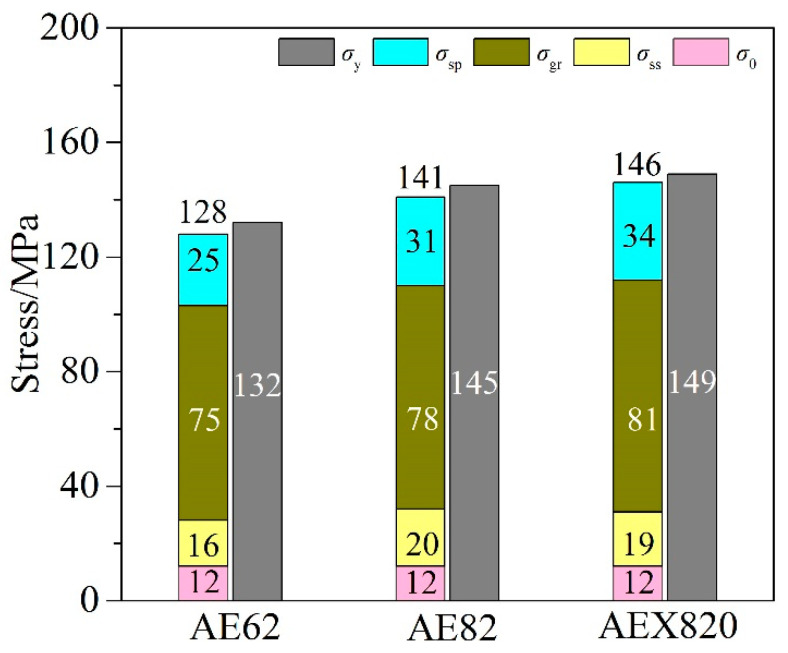
Contribution of different strengthening mechanisms to the YS of HPDC alloys at RT.

In this work, the secondary phase mainly consists of Mg_17_Al_12_ and Al_11_RE_3_ phases. Given that the added RE is a Ce-rich Ce-La rare earth mixture, the shear modulus of Al_11_Ce_3_ is used for approximate calculations. The area fractions of these second phases in the alloy were statistically analyzed to calculate the strengthening effect, with the material parameters summarized in Table 4. According to Equation (4), the *σ*_sp_ for these three alloys was estimated at 25 MPa, 31 MPa and 34 MPa, respectively. Then, based on Equations (1)–(4), the contribution of every strengthening to *σ*_y_ of HPDC alloys at RT is given in Figure 11. It is evident that the predominant strengthening mechanism in the studied alloys is *σ*_gr_, wherein fine grains predominate (Figure 4). The calculated *σ*_y_ is slightly lower than the experimental values, primarily due to the neglected strengthening effects of fine grains in the edge regions. The increase in Al addition enhances *σ*_gr_, *σ*_ss_ and *σ*_sp_, while the addition of Ca causes a slight reduction in *σ*_ss_, although it simultaneously increases *σ*_gr_ and *σ*_sp_.

Regarding ductility, the increasing addition of the alloying elements present dual effects: on one hand, the refined microstructure enhances the ductility; on the other hand, the coarser secondary phases easily formed in HPDC alloys are more prone to developing into crack initiation sites during tensile testing, resulting in reduced ductility [17,43]. Moreover, the defects formed in the HPDC alloys further decrease the elongation [43]. Consequently, the interaction between fine grains, coarse secondary phases and defects in HPDC alloys results in relatively lower ductility for AE82 and AEX820 alloys compared to AE62 alloys. For peak-aged samples, the lamellar- and rod-like β phases precipitate along the grain boundaries and within the grain interior of the alloys during T5 treatment (Figure 9). Similar to the other aged Mg alloys, these precipitates successfully enhance the strength but significantly decrease ductility associated with the increased phase concentration and possible defects like gas pores in the tensile bars [34,44].

**Figure 12:**
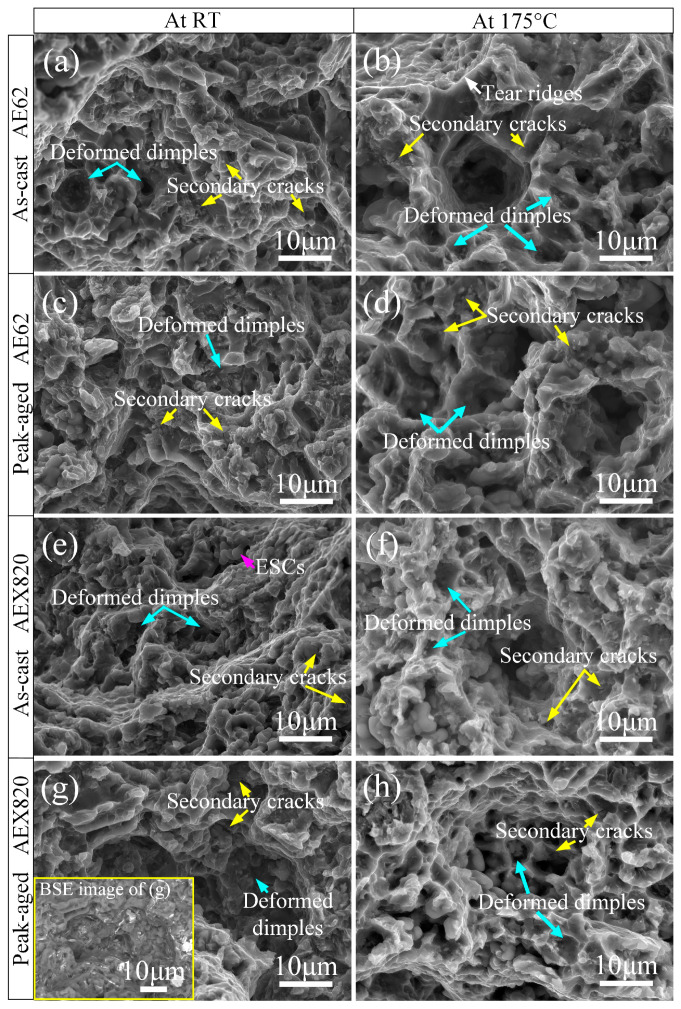
The fracture morphologies of both as-cast and peak-aged alloys (AE62 and AEX820) tensile-tested at RT and 175 °C. As-cast AE62 at RT (**a**) and 175 °C (**b**); Peak-aged AE62 at RT (**c**) and 175 °C (**d**); As-cast AEX820 at RT (**e**) and 175 °C (**f**); Peak-aged AEX820 at RT (**g**) and 175 °C (**h**). Note that the insertion is the BSE image of (**g**).

Figure 12 presents the typical fracture morphologies of AE62 and AEX820 alloys under different conditions (as-cast and peak-aged) tested at RT and 175 °C. For AE62 alloys tensile-tested at RT, the dimples (Figure 12a) are deeper than those of the peak-aged alloys (Figure 12c), indicating the superior ductility of the as-cast AE62 alloy compared to the peak-aged samples. A similar trend is observed when comparing the peak-aged AE62 alloy with the peak-aged AEX820 alloy under RT testing conditions. When the test temperature is increased to 175 °C, the dimples on the fracture surfaces of the samples are generally deeper and more numerous than the case at RT (Figure 12), suggesting that the ductility is relatively good at 175 °C (Table 3). This trend is consistent when comparing the peak-aged AE62 alloy with the peak-aged AEX820 alloy at 175 °C. Furthermore, the inset in Figure 12g shows a significant presence of secondary phases, indicating that these enriched secondary phases reduce the ductility of the alloy. Additionally, in the local region of the as-cast AEX820 alloy, few externally solidified crystals (ESCs) were identified, which are detrimental to the mechanical properties of high-pressure die-cast (HPDC) alloys [45]. The observed fracture features, including shallow deformed dimples, tear ridges and secondary cracks, indicate a mixed mode of cleavage and quasi-cleavage fracture [6]. Based on the findings reported above, the AE62 alloy demonstrates exceptional tensile properties among the studied HPDC Mg alloys. This promising outcome highlights the potential for the development of advanced HPDC Mg alloys. Therefore, further research is essential, particularly focusing on the incorporation of micro-alloying elements such as manganese (Mn), yttrium (Y) and calcium (Ca). These elements are expected to not only enhance the mechanical properties but also improve the corrosion resistance of the alloys, thereby broadening their applicability in various industrial sectors.

## 4. Conclusions

This study explored the microstructure and tensile properties of HPDC AE62, AE82 and AEX820 alloys under both as-cast and T5 aging conditions. The following conclusions can be drawn:

The HPDC alloys exhibited a composite microstructure characterized by a fine-grained edge region and an inner region of coarse grains rich in secondary phases, including Al_11_RE_3_ and Mg_17_Al_12_. The addition of a small amount of aluminum has an insignificant effect in refining the grain size of both the edge regions and the core regions, but it significantly influences the morphology and area fraction of the secondary phases. The average grain sizes of α-Mg in HPDC AE62, AE82 and AEX820 alloys are approximately 4.0 μm, 3.9 μm and 3.7 μm in the edge regions and about 13.9 μm, 12.8 μm and 12.1 μm, respectively, in the core regions, with the AEX820 alloy exhibiting the smallest average grain size of α-Mg. Increasing the Al concentration resulted in a morphological transition of the Mg_17_Al_12_ phase from net-broken-like in HPDC AE62 to semi-continuous-like in HPDC AE82 and AEX820 alloys. The minor addition of Ca introduced a dispersion of Al_2_Ca phases along dendritic boundaries. The increasing addition of Al and Ca promotes the continuous precipitation of β-Mg_17_Al_12_ within the grain interior, resulting in an enhanced aging–hardening response associated with the improvement in micro-hardness from 62.6 HV for peak-aged AE62 to 69.3 HV for the peak-aged AEX820 alloy.

Compared to HPDC AE62 alloy, the combined effects of increased Al and Ca additions and T5 aging treatment improved strength but significantly reduced ductility at both RT and 175 °C. The high YS observed in AE82 and AEX820 alloys at RT is attributed to solid-solution strengthening, secondary-phase strengthening and grain-refinement strengthening, with grain-refinement strengthening being the predominant factor. The significant reduction in E_f_ was primarily due to coarser secondary phases and possible casting defects. Considering a balance of strength, ductility and cost, the peak-aged AE62 alloys demonstrated optimal tensile properties, with UTS, YS and E_f_ values of ~241 MPa, ~141 MPa and ~7.1% at room temperature and ~129 MPa, ~96 MPa and ~19.8% at 175 °C, respectively. In contrast to the peak-aged AE62 alloy, the UTS and YS of peak-aged AEX820 alloys are improved by ~6.7% and ~14.2% at RT and ~8.5% and ~12.5% at 175 °C, respectively, while E_f_ decreased by 35.2% at RT and 33.3% at 175 °C, respectively, primarily due to the high area fraction of secondary phases. Further research, particularly focusing on the incorporation of micro-alloying elements such as manganese (Mn), yttrium (Y) and calcium (Ca), is expected to not only enhance the mechanical properties but also improve the corrosion resistance of the HPDC Mg alloys, thereby broadening their applicability in various industrial sectors.

## Figures and Tables

**Table 1:** The actual chemical compositions of the studied HPDC alloys (wt.%).

Alloy	Compositions (wt.%)				
	Al	Ce	La	Ca	Mg
AE62	5.723	1.152	0.632	0	Bal.
AE82	7.675	1.196	0.643	0	Bal.
AEX820	7.736	1.141	0.639	0.221	Bal.

**Table 2:** EDS point-scanning results on points (A–E) with the possible phases.

Point	Elemental Composition (at. %)					Possible Phases
	Al	Ce	La	Ca	Mg	
A	31.7	—	—	—	68.3	Mg_17_Al_12_
B	21.1	6.5	3.6	—	68.8	Al_2_RE
C	2.5	—	—	—	98.5	α-Mg
D	21.3	3.6	2.0	—	73.1	Al_11_RE_3_
E	29.7	—	—	1.1	69.2	Mg_17_Al_12_, Al_2_Ca

**Table 3:** The average tensile properties of as-cast and peak-aged alloys at RT and 175 °C.

Alloy	Condition	RT			175 °C		
		UTS/MPa	YS/MPa	E_f_/%	UTS/MPa	YS/MPa	E_f_/%
AE62	As-cast	$232_{-1}^{+7}$	$132_{-1}^{+2}$	$13.9_{-0.4}^{+0.5}$	$121_{-2}^{+4}$	$90_{-3}^{+2}$	$27.5_{-0.5}^{+1.2}$
	Peak-aged	$241_{-2}^{+4}$	$141_{-2}^{+6}$	$7.1_{-0.3}^{+0.7}$	$129_{-2}^{+1}$	$96_{-2}^{+2}$	$19.8_{-0.5}^{+0.8}$
AE82	As-cast	$243_{-1}^{+2}$	$145_{-2}^{+2}$	$9.8_{-0.2}^{+0.4}$	$127_{-3}^{+1}$	$98_{-2}^{+3}$	$23.2_{-0.3}^{+0.8}$
	Peak-aged	$254_{-2}^{+2}$	$155_{-3}^{+1}$	$5.6_{-0.1}^{+0.2}$	$138_{-2}^{+3}$	$105_{-3}^{+4}$	$14.3_{-0.3}^{+0.5}$
AEX820	As-cast	$247_{-3}^{+4}$	$149_{-2}^{+5}$	$8.2_{-0.4}^{+0.2}$	$132_{-1}^{+2}$	$102_{-2}^{+1}$	$21.6_{-0.6}^{+0.5}$
	Peak-aged	$257_{-1}^{+2}$	$161_{-4}^{+2}$	$4.6_{-0.2}^{+0.2}$	$140_{-3}^{+2}$	$108_{-3}^{+1}$	$13.2_{-0.5}^{+0.6}$

**Table 4:** Mean values for the elastic constants of Mg [35] and second phases [35].

Phase	Shear Modulus, *μ*	Poisson’s Ratio, *ν*
Mg	17.2	0.35
Mg_17_Al_12_	24	0.21
Al_11_Ce_3_	47.2	0.21

## Data Availability

The original contributions presented in the study are included in the article, further inquiries can be directed to the corresponding authors.
